# Latent Profile Analysis of the Five Facet Mindfulness Questionnaire in a Sample With a History of Recurrent Depression

**DOI:** 10.1177/1073191117715114

**Published:** 2017-06-19

**Authors:** Jenny Gu, Anke Karl, Ruth Baer, Clara Strauss, Thorsten Barnhofer, Catherine Crane

**Affiliations:** 1 University of Sussex, Falmer, East Sussex, UK; 2 University of Exeter, Exeter, Devon, UK; 3 University of Kentucky, Lexington, KY, USA; 4 University of Oxford, Oxford, Oxfordshire, UK

**Keywords:** mindfulness, Five Facet Mindfulness Questionnaire, latent profile analysis, depression, self-compassion

## Abstract

Extending previous research, we applied latent profile analysis in a sample of adults with a history of recurrent depression to identify subgroups with distinct response profiles on the Five Facet Mindfulness Questionnaire and understand how these relate to psychological functioning. The sample was randomly divided into two subsamples to first examine the optimal number of latent profiles (test sample; *n* = 343) and then validate the identified solution (validation sample; *n* = 340). In both test and validation samples, a four-profile solution was revealed where two profiles mapped broadly onto those previously identified in nonclinical samples: “high mindfulness” and “nonjudgmentally aware.” Two additional subgroups, “moderate mindfulness” and “very low mindfulness,” were observed. “High mindfulness” was associated with the most adaptive psychological functioning and “very low mindfulness” with the least adaptive. In most people with recurrent depression, mindfulness skills are expressed evenly across different domains. However, in a small minority a meaningful and replicable uneven profile indicating nonjudgmental awareness is observable. Current findings require replication and future research should examine the extent to which profiles change from periods of wellness to illness in people with recurrent depression and how profiles are influenced by exposure to mindfulness-based intervention.

Mindfulness can be conceptualized as an innate psychological capacity which arises from the intentional direction of attention to present moment experience in a curious, nonjudgmental, and accepting way. A number of definitions exist but most share similarities in their descriptions of the “what” and the “how” of mindfulness as a dispositional characteristic (Baer, in press). To enhance understanding of the nature of mindfulness, its expression in different individuals, and its consequences for psychological health, it is critical that it can be operationalized and measured reliably. The Five Facet Mindfulness Questionnaire (FFMQ) is currently one of the most comprehensive and widely used measures of dispositional mindfulness (Quaglia, Braun, Freeman, McDaniel, & Brown, 2016). Derived from a factor analysis of items from a number of existing mindfulness scales, the FFMQ has five facets intended to capture the key aspects of mindfulness as a dispositional variable, labelled observing, describing, acting with awareness, nonreactivity, and nonjudging (Baer et al., 2008; Baer, Smith, Hopkins, Krietemeyer, & Toney, 2006).

Interestingly, both early validation work and later research has shown that while the describing, acting with awareness, nonreactivity, and nonjudging subscales consistently load on to a higher order mindfulness factor, the observing facet functions differently in different populations. Specifically, in samples of nonmediators the observing subscale does not consistently correlate positively and significantly with other mindfulness facets (e.g., Baer et al., 2006; Brown, Bravo, Roos, & Pearson, 2015; Fernandez, Wood, Stein, & Rossi, 2010; Gu et al., 2016) or load on to the higher order mindfulness factor (Baer et al., 2008), whereas in samples with meditation experience the expected relations between observing and the other facets of mindfulness are typically seen (e.g., Gu et al., 2016).

Although many studies treat mindfulness as a unidimensional construct, these findings draw attention to the importance of considering both how the psychological skills that together comprise the overarching construct of mindfulness develop, relate to, and interact with one another and how they act independently and collectively to promote positive psychological states. These issues can be considered either by examining the predictive value of the various FFMQ facets and their interactions in relation to external indicators of psychological functioning, or by examining the way in which different facets of mindfulness present themselves within individuals.

Taking the former approach, Eisenlohr-Moul, Walsh, Charnigo, Lynam, and Baer (2012) examined the relationship between facets of dispositional mindfulness and alcohol and tobacco use in students. Results showed that increased observing was associated with reduced alcohol use when levels of nonreactivity were 1 standard deviation above the mean, but were associated with increased alcohol use when levels of nonreactivity were 1 standard deviation below the mean. Similar findings were also observed for tobacco use. Likewise, a study of treatment seeking adults with *Diagnostic and Statistical Manual of Mental Disorders–Fourth edition* (*DSM-IV*) anxiety and/or depressive disorders showed that nonreactivity was a significant moderator of the association between observing and depression symptoms (but not anxiety symptoms), such that only individuals with very low levels of nonreactivity showed a positive relationship between observing and depressive symptoms (Desrosiers, Vine, Curtiss, & Klemanski, 2014). In the same study, individuals with lower levels of nonreactivity and higher levels of observing also showed more rumination and worry and less reappraisal. Finally, Tomfohr, Pung, Mills, and Edwards (2012) showed that in healthy young adults, in regression analyses predicting levels of the inflammatory marker (Interleukin-6), there was a significant interaction between observing and nonreactivity facets of the FFMQ. Specifically, in individuals with nonreactivity scores 1 standard deviation above the mean, there was a significant association between observing and lower Interleukin-6, which was attenuated in individuals with nonreactivity scores 1 standard deviation below the mean, and no longer significant in individuals 2 standard deviations below the mean. Together, the above findings illustrate the potential importance of considering the interactions between mindfulness facets for psychological health and well-being and suggest that focusing either on the relationship between overall dispositional mindfulness score, or isolated individual facets, and measures of psychological health may mask important, more nuanced associations. However, the relative importance of looking at facet scores versus total scores on the FFMQ rests in part on the degree to which subgroups, characterized by high scores on some facets of the FFMQ alongside low scores on others, actually exist in populations of interest.

Likewise, it might be expected that such uneven profiles on the FFMQ would be observed alongside more flat profiles. For example, mindfulness skills training programs are often based on the premise that in cultivating the overall capacity of mindful awareness, acquisition of particular early skills (such as stabilization of attention) provides an important foundation for later skill development (e.g., the capacity to relate to difficult emotional states in a nonreactive way, e.g., mindfulness-based cognitive therapy [MBCT]; Segal, Williams, & Teasdale, 2013). Thus, while different aspects of mindfulness are regarded as mutually reinforcing, during mindfulness training, capacities assessed by some facets of the FFMQ might be expected to increase prior to others. It is also possible that in people who have never received mindfulness training, different mindfulness capacities may develop asynchronously and/or be manifest to different degrees as a result of particular neurodevelopmental or temperamental factors, or because the presence of particular psychological disorders disrupts some capacities (e.g., attentional control) while leaving others (e.g., the capacity to recognize and label emotions) intact. In either case, uneven profiles might be observed for some people, in some contexts, or at some times.

Recently, initial steps to explore the issue of response profiles on the FFMQ have been taken. Using latent profile analysis (LPA), Pearson, Lawless, Brown, and Bravo (2015) and Bravo, Boothe, and Pearson (2016) explored subgroups characterized by different profiles on the five facets of the FFMQ in college student samples. Pearson et al. (2015) identified four subgroups, which they termed the “high mindfulness” group, “low mindfulness” group, “judgmentally observing” group, and “nonjudgmentally aware” group. The judgmentally observing group (characterized by very low scores on nonjudging and acting with awareness subscales, moderate scores on the describing subscale, and relatively high scores on the observing subscale) and the low mindfulness group (characterized by relatively low scores on all FFMQ subscales) showed the poorest psychological well-being, while the high mindfulness group (relatively high scores on all FFMQ subscales) and the nonjudgmentally aware group (relatively low scores on observing and high scores on nonjudging and acting with awareness facets) showed the greatest well-being.

Bravo et al. (2016) subsequently confirmed the presence of the same four subgroups in a second college student sample composed of those with and without any meditation experience. Interestingly, the proportion of students falling into each group differed between the nonmeditator (NM) and meditator (M) groups: “high mindfulness” group (NM = 17.34%; M = 27.94%), “low mindfulness” group (NM = 54.85%; M = 59.72%), “judgmentally observing” group (NM = 12.87%; M = 5.50%), and “nonjudgmentally aware” group (NM = 14.94%; M = 6.84%), but the four-class solution remained the most parsimonious both across the whole sample and in the two subgroups. These results suggest that a majority of individuals have a relatively flat profile of mindfulness skills across the five facets of the FFMQ, but that a significant minority (just under a third of those without meditation experience) do show uneven profiles, with latent profiles membership appearing to be meaningfully related to mental well-being.

The above findings raise the question of whether the same four latent profiles would be observed in samples with a clinical history, at risk of psychological distress and dysfunction, and if so, whether the low mindfulness and judgmentally observing profiles, identified as associated with poorer psychological well-being in college students, would be more prevalent in such groups. This knowledge would have important implications both for clinical research and assessment—in particular, concerning the utility of quantifying level of dispositional mindfulness based on a single sum-score, and clinical practice—for example, informing the extent to which programs for skill acquisition might be personalized to an individual’s baseline profile of strengths and weaknesses in mindfulness, where uneven profiles are observed. In addition, if individuals in at-risk samples are found to frequently display uneven profiles this would have implications for our understanding of the underlying construct of mindfulness and its expression.

In this article, we explored latent profiles on the FFMQ in individuals at risk of depressive relapse using baseline data from participants enrolled in two clinical trials of MBCT for prevention of depressive relapse that shared common inclusion criteria (Kuyken et al., 2015; J. M. G. Williams, Crane, et al., 2014). In both trials, data were gathered from participants at the point of entry to the study, prior to any exposure to a mindfulness-based intervention. In both samples, participants were eligible if they had experienced three or more prior episodes of depression, were currently in remission or partial remission from depression, and were not receiving regular ongoing psychotherapy. Comorbid disorders rendering individuals ineligible for participation were almost identical across the two trials and both had populations with similar sociodemographic characteristics. In both cases, participants were recruited through self-referral and primary care database searches, with one requiring maintenance antidepressant medication at entry and one leaving this unrestricted.

We pooled data across these trials and the overall sample was then randomly divided into two subsamples to first examine the optimal number of latent profiles (test sample) and then validate the identified profile solution (validation sample). It was decided a priori that the test sample would be the larger of the two subsamples. We were interested in exploring: (a) whether the same latent profiles would be observed and replicated in an at-risk sample with a history of recurrent depression as in the previous studies with college students and (b) how constructs linked to mindfulness (self-compassion, residual depressive symptoms) and clinical history would relate to each of the latent profiles observed. We hypothesized that profiles characterized by low levels of mindfulness across the different facets would be more evident in our sample than the college student samples studied previously, due to the clinical history of our participants. However, we additionally hypothesized that where the profiles observed in our sample were similar to those identified in previous research, relationships with external indicators of psychological functioning would be comparable.

## Method

### Participants

The sample consisted of participants from two trials that examined the effectiveness of MBCT compared with control conditions at reducing relapse to depression in people with recurrent major depressive disorder in remission (Preventing depressive relapse in NHS settings through MBCT [PREVENT] trial; Kuyken et al., 2015; and Staying Well After Depression [SWAD] trial; J. M. G. Williams, Crane, et al., 2014). Both PREVENT and SWAD were multicenter trials, with PREVENT recruiting from general practices in rural and urban settings in the United Kingdom and SWAD recruiting from the community, primary care, and mental health clinics in the regions of Oxford, England, and Bangor, North Wales. Inclusion criteria for both trials were (a) a diagnosis of recurrent major depressive disorder in full or partial remission according to the *DSM-IV* (American Psychiatric Association, 1994), (b) three or more previous depressive episodes, and (c) being 18 years or older. Exclusion criteria from both trials were having (a) a current major depressive episode; (b) a comorbid diagnosis of current substance misuse, organic brain damage, current or past psychosis, current or past bipolar disorder, persistent antisocial behavior, or persistent self-harm requiring clinical management or therapy; and (c) formal concurrent psychotherapy.

In this article, we used data from baseline measurement, prior to any exposure to MBCT. Participants were included unless they had missing data for composite scores on all five facets. A total of 683 participants fit the criteria (97.85% of the total number of participants randomized to PREVENT and SWAD); 410 participants from the PREVENT trial and 273 participants from the SWAD trial. This overall sample was randomly divided into the test sample (*n* = 343) and validation sample (*n* = 340) using the random sampling function in SPSS.

The test sample comprised 252 women (73.5%) and 91 men (26.5%). The mean age was 46.74 years (*SD* = 13.03; range = 18-79 years) and most of the sample was White (97.1%). In terms of educational qualifications, 16 (4.7%) had no qualifications, 64 (18.7%) had some General Certificate of Secondary Education/O Levels, 95 (27.7%) had some A Levels or comparable vocational qualifications, 74 (21.6%) had a bachelor’s degree, 29 (8.5%) had a master’s degree, and 54 (15.7%) had a doctoral degree or professional qualification. Four participants had other qualifications and data on education were missing for seven. The validation sample consisted of 258 women (75.9%) and 82 men (24.1%). Mean age was 47.36 years (*SD* = 12.03; range = 18-74 years). Most of the sample was White (97.9%). Eleven (3.2%) had no qualifications, 60 (17.6%) had some General Certificate of Secondary Education/O Levels, 119 (35.0%) had some A Levels or comparable vocational qualifications, 68 (20.0%) had a bachelor’s degree, 31 (9.1%) had a master’s degree, and 37 (10.9%) had a doctoral degree or professional qualification. Four participants had other qualifications and data on education were missing for 10.

### Measures

#### Five Facet Mindfulness Questionnaire

The 39-item version of the FFMQ (Baer et al., 2006) measures levels of dispositional mindfulness in everyday life across five facets: observing, describing, acting with awareness, nonjudging, and nonreactivity. Items are rated on a 5-point Likert-type scale ranging from 1 (*never or very rarely true*) to 5 (*very often or always true*). Sample items from each facet include “I notice the smells and aromas of things” (observing), “I’m good at finding words to describe my feelings” (describing), “I find myself doing things without paying attention” (acting with awareness, negatively phrased), “I disapprove of myself when I have illogical ideas” (nonjudging, negatively phrased), and “When I have distressing thoughts or images, I am able just to notice them without reacting” (nonreactivity). Following Pearson et al. (2015), facet scores were computed by calculating the means of relevant item scores, rather than summing item scores. Mean scores on FFMQ facets did not significantly differ across test and validation samples: observing (*M*_test_ = 3.08, *SD*_test_ = 0.74; *M*_validation_ = 3.09, *SD*_validation_ = 0.67), describing (*M*_test_ = 3.24, *SD*_test_ = 0.80; *M*_validation_ = 3.24, *SD*_validation_ = 0.81), acting with awareness (*M*_test_ *=* 3.01, *SD*_test_ = 0.65; *M*_validation_ = 2.98, *SD*_validation_ = 0.65), nonjudging (*M*_test_ = 3.05, *SD*_test_ = 0.75; *M*_validation_ = 3.07, *SD*_validation_ = 0.77), and nonreactivity (*M*_test_ = 2.84, *SD*_test_ = 0.68; *M*_validation_ = 2.81, *SD*_validation_ = 0.61). Negatively phrased items were reverse-scored prior to analysis. Cronbach’s alpha for each facet in the test sample were .80 (observing), .88 (describing), .84 (acting with awareness), .85 (nonjudging), and .81 (nonreactivity). Cronbach’s alpha for each facet in the validation sample were .74 (observing), .89 (describing), .83 (acting with awareness), .85 (nonjudging), and .77 (nonreactivity).

#### Self-Compassion Scale (SCS)

The SCS (Neff, 2003) is a 26-item self-report instrument. Each item is rated on a 5-point Likert-type scale ranging from 1 (*almost never*) to 5 (*almost always*) and the measure yields both a total score and scores on six subscales: self-kindness, self-judgement, common humanity, isolation, mindfulness, and overidentification. Higher scores indicate higher levels for each respective scale. Although the six-factor hierarchical structure of the scale has been called into question (e.g., Strauss et al., 2016; M. J. Williams et al., 2014), researchers are encouraged to continue analyzing subscale scores and the total SCS score (Neff, 2016). Composite scale and subscale scores were computed by calculating the means of relevant item scores. Mean total scale and subscale scores did not significantly differ across test and validation samples: total SCS (*M*_test_ = 2.53, *SD*_test_ = 0.60; *M*_validation_ = 2.51, *SD*_validation_ = 0.59), self-kindness (*M*_test_ = 2.46, *SD*_test_ = 0.78; *M*_validation_ = 2.45, *SD*_validation_ = 0.81), self-judgement (*M*_test_ = 2.33, *SD*_test_ = 0.80; *M*_validation_ = 2.30, *SD*_validation_ = 0.78), common humanity (*M*_test_ = 2.85, *SD*_test_ = 0.86; *M*_validation_ = 2.84, *SD*_validation_ = 0.90), isolation (*M*_test_ = 2.34, *SD*_test_ = 0.81; *M*_validation_ = 2.29, *SD*_validation_ = 0.76), mindfulness (*M*_test_ = 2.90, *SD*_test_ = 0.75; *M*_validation_ = 2.89, *SD*_validation_ = 0.80), and overidentification (*M*_test_ = 2.31, *SD*_test_ = 0.83; *M*_validation_ = 2.27, *SD*_validation_ = 0.73). Negatively phrased items were reverse-scored prior to analysis. Cronbach’s alpha for the total scale and subscales in the test sample were .91 (total SCS), .78 (self-kindness), .79 (self-judgement), .75 (common humanity), .73 (isolation), .70 (mindfulness), and .76 (overidentification). Cronbach’s alpha for the total scale and subscales in the validation sample were .91 (total SCS), .82 (self-kindness), .80 (self-judgement), .79 (common humanity), .69 (isolation), .78 (mindfulness), and .66 (overidentification).

#### Beck Depression Inventory–Second Edition (BDI-II)

The BDI-II (Beck, Steer, & Brown, 1996) is a 21-item measure used to assess the severity of depressive symptomatology. Each item relates to a symptom of depression, and provides four response options, each describing an increasingly severe presentation of the symptom. Each item is scored on a 4-point scale ranging from 0 (*not at all*) to 3 (*extreme form of each symptom*). Items are summed to give a single total score, which ranges from 0 to 63; a score of 0 to 13 is considered to reflect minimal depression, 14 to 19 mild depression, 20 to 29 moderate depression, and 30 to 63 severe depression. Mean total scores for BDI-II did not differ significantly across test (*M* = 11.48, *SD* = 9.44) and validation samples (*M* = 11.97, *SD* = 9.98). Cronbach’s alpha for total BDI-II was .92 in both samples.

#### Sociodemographic and Clinical History

Sociodemographic variables (age, sex, ethnicity, educational level) and clinical history variables (age of onset of first episode of major depression, number of episodes of major depression) were derived from the Structured Clinical Interview for *DSM-IV* (First, Spitzer, Gibbon, & Williams, 1995) and associated sociodemographic information questions conducted with each participant at entry to the PREVENT or SWAD trial. There were no significant differences between test and validation samples on all sociodemographic and clinical history variables.

### Statistical Analyses

LPAs were conducted using maximum likelihood estimation with robust standard errors in M*plus* version 7.4 (Muthén & Muthén, 1998-2015). To maintain parsimony, latent profile models containing one to a maximum of seven profiles were fit to the data. To determine the optimal number of latent profiles in our test and validation samples, each model was assessed using the following fit indices: the Akaike information criterion (AIC; Akaike, 1974), Bayesian information criterion (BIC; Schwartz, 1978), sample-size-adjusted BIC (sBIC; Yang, 2006), bootstrapped likelihood ratio test (BLRT; McLachlan & Peel, 2000), and Lo–Mendell–Rubin adjusted likelihood ratio test (LMR-LRT; Lo, Mendell, & Rubin, 2001). Both the BLRT and LMR-LRT evaluate whether a *k* profile model fits significantly better compared with a *k*-1 profile model. Smaller AIC, BIC, and sBIC values indicate better model fit. Of the likelihood-based tests, the BLRT was found to be the better indicator of the appropriate number of profiles and of the information criterion indices, the BIC has been found to be a superior indicator (Nylund, Asparouhov, & Muthén, 2007). In addition to using fit indices to determine the optimal profile solution for the test and validation samples, we examined probabilities of group classification based on the most likely profile membership (posterior classification probabilities) for competing models. Posterior classification probabilities range from 0 to 1, with higher diagonal values (in a matrix of probabilities for the most likely latent profile membership by latent profile) indicating greater confidence for the model. Following Bravo et al. (2016) and Pearson et al. (2015) and to facilitate the labelling of emerging profiles, facet means were standardized so that positive values are greater than the mean and negative values are below the mean.

On determining the optimal number of latent groups in both test and validation samples, mean facet scores within profiles across the two samples were compared using independent *t* tests and Cohen’s *d* effect sizes, to examine any differences between profile solutions across the samples. The relationships between profiles and constructs related to mindfulness (SCS and subscales, depressive symptoms, and clinical history variables) were also examined for each sample. This was achieved by using the auxiliary variable function in M*plus* (the “Bolck, Croon, and Hagenaars” method, or the “BCH” method; Bolck, Croon, & Hagenaars, 2004), which tests the equality of the means of mindfulness-related variables across the latent profiles (mean differences across profiles) using Wald chi-square tests. This function allows the relationships between profiles and other auxiliary variables to be explored without directly including these variables in the model, which could distort the model in the sense that it would not solely be defined by the latent profile variables (i.e., FFMQ facets). The BCH method is the most robust approach and the recommended method for examining relationships between profiles and continuous variables (Asparouhov & Muthén, 2014; Bakk & Vermunt, 2016).

## Results

### Latent Profile Analysis

#### Test Sample

Table 1 reports the fit indices for the latent profile models containing one to seven profiles fit to data from the test sample. The BLRT results were significant for the four-profile versus three-profile comparison and the six-profile versus five-profile comparison, but nonsignificant for the five-profile versus four-profile comparison and the seven-profile versus six-profile comparison. This indicates preference for the four-profile and six-profile solutions. The BIC value was smallest for the four-profile model. The LMR-LRT showed that a four-profile model fit significantly better than a three-profile model and a two-profile model fit significantly better than a one-profile solution. The sBIC and AIC values were smallest for the seven-profile model, but continue to decrease past this model. On the whole, and given that the BIC and BLRT have been found to be superior indicators of the number of profiles compared with other information criterion indices and likelihood-based tests (Nylund et al., 2007), the four-profile model appeared to be the optimal solution for the current sample. Additionally, posterior classification probabilities were greater for the four-profile model (.81 and above) compared with the six-profile model (.65 and above).

**Table 1.** Fit Indices for Models Containing One to Seven Latent Profiles in the Test Sample (*n* = 343).

Fit indices	Number of profiles						
	1	2	3	4	5	6	7
AIC	4861.42	4700.19	4653.94	4601.77	4593.51	4583.12	4574.12
BIC	4899.80	4761.59	4738.37	4709.23	4724.00	4736.63	4750.66
sBIC	4868.07	4710.84	4668.58	4620.41	4616.14	4609.74	4604.73
Entropy	—	0.623	0.678	0.746	0.776	0.725	0.749
LMR-LRT	—	2 vs. 1; value = 168.42; *p* < .007	3 vs. 2; value = 56.64; *p* = .214	4 vs. 3; value = 62.38; *p* = .030	5 vs. 4; value = 19.70; *p* = .256	6 vs. 5; value = 21.77; *p* = .299	7 vs. 6; value = 20.42; *p* = .557
BLRT	—	2 vs. 1; *p* < .001	3 vs. 2; *p* < .001	4 vs. 3; *p* < .001	5 vs. 4; *p* = .133	6 vs. 5; *p* = .030	7 vs. 6; *p* = .140
*n* in each profile	P1 = 343	P1 = 144; P2 = 199	P1 = 177; P2 = 132; P3 = 34	P1 = 101; P2 = 32; P3 = 193; P4 = 17	P1 = 8; P2 = 17; P3 = 102; P4 = 185; P5 = 31	P1 = 33; P2 = 136; P3 = 10; P4 = 15; P5 = 124; P6 = 25	P1 = 40; P2 = 130; P3 = 10; P4 = 11; P5 = 120; P6 = 7; P7 = 25

*Note*. AIC = Akaike information criterion; BIC = Bayesian information criterion; sBIC = sample-size-adjusted BIC; LMR-LRT = Lo–Mendell–Rubin adjusted likelihood ratio test; BLRT = bootstrapped likelihood ratio test; FFMQ = Five Facet Mindfulness Questionnaire. Values are based on running models which specified standardized FFMQ facet scores.

**Figure 1.**
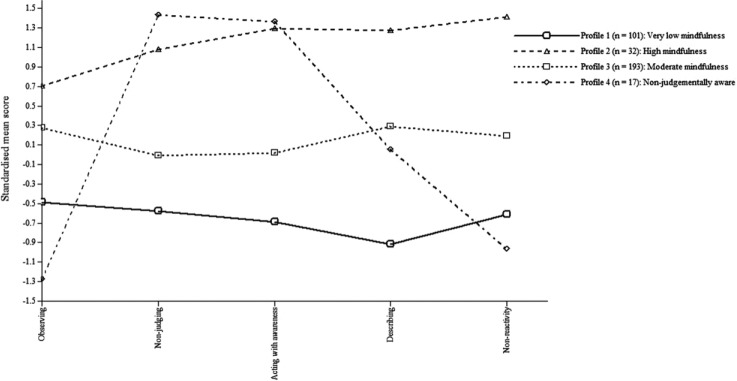
Plot of the standardized mean scores on mindfulness facets across the four latent profiles in the test sample.

The four-profile model had a medium-high entropy value of 0.75 (Clark & Muthén, 2009), which indicates that 75% of participants were correctly classified in the appropriate latent profile. Table 2 presents the mean FFMQ facet scores across the four latent profiles and Figure 1 provides a visual illustration of this. Profile 1 comprises 29.45% of the test sample (*n* = 101) and was labelled the “very low mindfulness” group due to their low mean score on every FFMQ facet, with most standardized scores between 0.5 and 1 standard deviation below the mean (*z* = −0.49 to −0.92; cf. standardized facet means between 0 and −0.5 for the “low mindfulness” profile in Bravo et al., 2016; Pearson et al., 2015). Profile 2 contained 9.33% of the sample (*n* = 32) and was labelled the “high mindfulness” group due to their relatively high mean score on every FFMQ facet, with most standardized scores over 1 standard deviation above the mean (*z* = 0.71 to 1.42). Profile 3 consisted of 56.27% of the sample (*n* = 193) and was labelled the “moderate mindfulness” group because facet scores were close to the mean (*z* = −0.01 to 0.29). Profile 4 contained 4.96% of the sample (*n* = 17) and was labelled the “nonjudgmentally aware” group because they had high mean scores on the nonjudging (*z* = 1.44) and acting with awareness facets (*z* = 1.36), but low mean scores on the observing facet (*z* = −1.28).

**Table 2.** Mean Scores on Mindfulness Facets Across Latent Profiles in the Test Sample (*N* = 343).

	Profile 1: Very low mindfulness (*n* = 101)	Profile 2: High mindfulness (*n* = 32)	Profile 3: Moderate mindfulness (*n* = 193)	Profile 4: Nonjudgmentally aware (*n* = 17)
	*M* (*SE*, variance)	*M* (*SE*, variance)	*M* (*SE*, variance)	*M* (*SE*, variance)
Mindfulness facets (*standardized* scores^a^)				
Observing	−0.49 (0.14, 0.75)	0.71 (0.24, 0.75)	0.28 (0.14, 0.75)	−1.28 (0.21, 0.75)
Describing	−0.92 (0.15, 0.54)	1.28 (0.14, 0.54)	0.29 (0.18, 0.54)	0.06 (0.30, 0.54)
Acting with awareness	−0.69 (0.21, 1.30)	1.30 (0.30, 0.59)	0.02 (0.09, 0.59)	1.36 (0.32, 0.59)
Nonjudging	−0.58 (0.18, 0.67)	1.08 (0.29, 0.67)	−0.01 (0.09, 0.67)	1.44 (0.30, 0.67)
Nonreactivity	−0.62 (0.16, 0.62)	1.42 (0.20, 0.62)	0.19 (0.12, 0.62)	−0.97 (0.26, 0.62)
Mindfulness facets (*unstandardized* scores)				
Observing	2.72 (0.10, 0.41)	3.60 (0.18, 0.41)	3.28 (0.11, 0.41)	2.14 (0.15, 0.41)
Describing	2.51 (0.12, 0.34)	4.26 (0.11, 0.34)	3.47 (0.14, 0.34)	3.28 (0.24, 0.34)
Acting with awareness	2.56 (0.14, 0.25)	3.85 (0.19, 0.25)	3.02 (0.06, 0.25)	3.89 (0.21, 0.25)
Nonjudging	2.62 (0.13, 0.37)	3.86 (0.21, 0.37)	3.05 (0.07, 0.37)	4.12 (0.22, 0.37)
Nonreactivity	2.43 (0.11, 0.28)	3.80 (0.14, 0.28)	2.98 (0.08, 0.28)	2.19 (0.18, 0.28)

*Note. SE* = standard error of the mean.

a Scores have been standardized so that positive values are above the mean and negative values are below the mean.

#### Validation Sample

Table 3 reports the fit indices for the latent profile models containing one to seven profiles fit to data from the validation sample. The BIC value was smallest for the four-profile model. The BLRT showed that a five-profile model fit significantly better than a four-profile model, but a six-profile model did not fit significantly better than a five-profile model. The LMR-LRT showed that a two-profile model fit significantly better than a one-profile solution, and all other comparisons were nonsignificant. The sBIC and AIC values were smallest for the seven-profile model, but continue to decrease past the seven-profile model. On the whole, given that the BIC and BLRT have been found to be superior indicators of the number of profiles (Nylund et al., 2007), the four-profile or five-profile model appeared to be the optimal solution for the current sample. Inspection of the posterior classification probabilities showed that they were greater for the four-profile model (.74 and above) compared with the five-profile model (.61 and above). Taken together, we decided to settle for the four-profile solution in the validation sample.

**Table 3.** Fit Indices for Models Containing One to Seven Latent Profiles in the Validation Sample (*n* = 340).

Fit indices	Number of profiles						
	1	2	3	4	5	6	7
AIC	4810.34	4672.84	4635.98	4608.26	4598.75	4591.35	4584.21
BIC	4848.63	4734.10	4720.22	4715.47	4728.94	4744.51	4760.34
sBIC	4816.90	4683.35	4650.43	4626.65	4621.08	4617.62	4614.42
Entropy	—	0.627	0.692	0.734	0.734	0.711	0.726
LMR-LRT	—	2 vs. 1; value = 145.34; *p* = .002	3 vs. 2; value = 47.50; *p* = .079	4 vs. 3; value = 38.62; *p* = .094	5 vs. 4; value = 20.91; *p* = .686	6 vs. 5; value = 18.86; *p* = .211	7 vs. 6; value = 18.61; *p* = .561
BLRT	—	2 vs. 1; *p* < .001	3 vs. 2; *p* < .001	4 vs. 3; *p* < .001	5 vs. 4; *p* = .020	6 vs. 5; *p* = .095	7 vs. 6; *p* = .080
*n* in each profile	P1 = 340	P1 = 224; P2 = 116	P1 = 242; P2 = 44; P3 = 54	P1 = 29; P2 = 204; P3 = 56; P4 = 51	P1 = 56; P2 = 194; P3 = 24; P4 = 51; P5 = 15	P1 = 6; P2 = 71; P3 = 77; P4 = 38; P5 = 138; P6 = 10	P1 = 97; P2 = 12; P3 = 35; P4 = 66; P5 = 5; P6 = 7; P7 = 118

*Note.* AIC = Akaike information criterion; BIC = Bayesian information criterion; sBIC = sample-size-adjusted BIC; LMR-LRT = Lo–Mendell–Rubin adjusted likelihood ratio test; BLRT = bootstrapped likelihood ratio test; FFMQ = Five Facet Mindfulness Questionnaire. Values are based on running models which specified standardized FFMQ facet scores.

**Figure 2.**
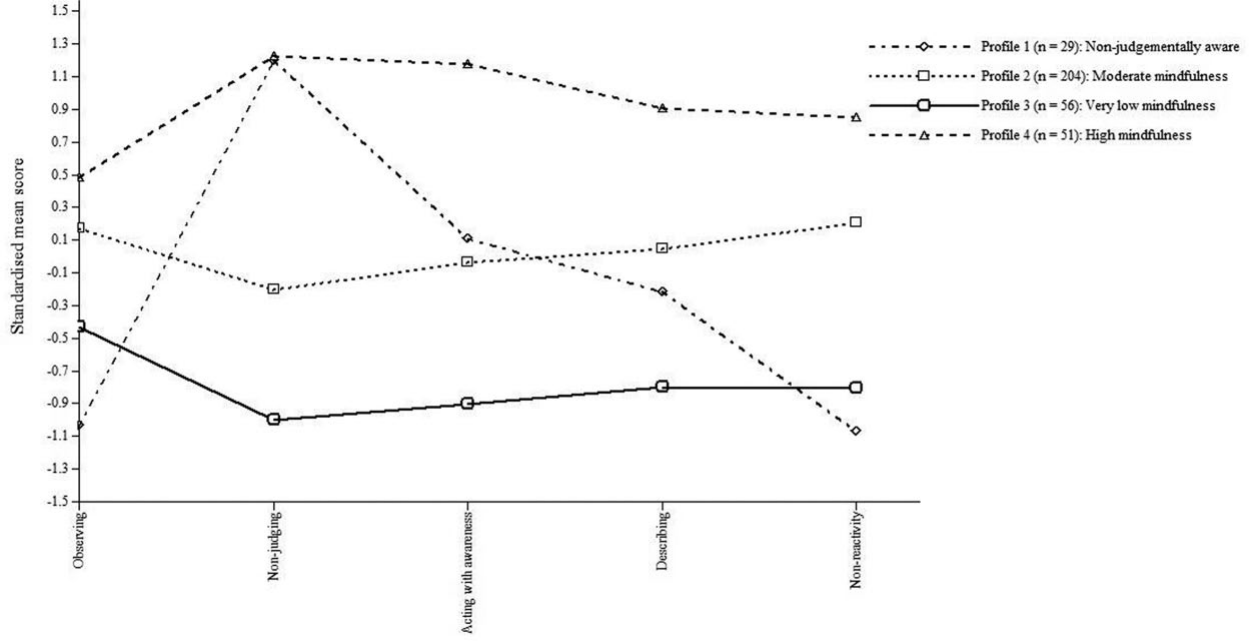
Plot of the standardized mean scores on mindfulness facets across the four latent profiles in the validation sample.

The four-profile model had a medium-high entropy value of 0.73 (Clark & Muthén, 2009), which indicates that 73% of participants were correctly classified in the appropriate latent profile. Table 4 presents the mean FFMQ facet scores across the four latent profiles and Figure 2 provides a visual illustration of this. The four profiles in the validation sample supported those identified in the test sample. Profile 1 comprises 8.53% of the validation sample (*n* = 29) and was labelled the “nonjudgmentally aware” group because they had high mean scores on the nonjudging facet (*z* = 1.20) and moderate scores above the mean on the acting with awareness facet (*z* = 0.11), but scores below the mean on all other facets (*z* = −0.22 to −1.07). Profile 2 consisted of 60% of the sample (*n* = 204) and was labelled the “moderate mindfulness” group because all facet scores were close to the mean (*z* = −0.20 to 0.20). Profile 3 contained 16.47% of the sample (*n* = 56) and was labelled the “very low mindfulness” group due to their low mean score on every FFMQ facet, with most standardized scores between 0.5 and 1 standard deviation below the mean (*z* = −0.43 to −1.00; cf. standardized facet means between 0 and −0.5 for the “low mindfulness” profile in Bravo et al., 2016; Pearson et al., 2015). Profile 4 comprises 15% of the sample (*n* = 51) and was labelled the “high mindfulness” group due to their relatively high mean score on every FFMQ facet, with most standardized scores close to, or over, 1 standard deviation above the mean (*z* = 0.48 to 1.23).

**Table 4.** Mean Scores on Mindfulness Facets Across Latent Profiles in the Validation Sample (*N* = 340).

	Profile 1: Nonjudgmentally aware (*n* = 29)	Profile 2: Moderate mindfulness (*n* = 204)	Profile 3: Very low mindfulness (*n* = 56)	Profile 4: High mindfulness (*n* = 51)
	*M* (*SE*, variance)	*M* (*SE*, variance)	*M* (*SE*, variance)	*M* (*SE*, variance)
Mindfulness facets (*standardized* scores^a^)				
Observing	−1.03 (0.23, 0.82)	0.18 (0.09, 0.82)	−0.43 (0.21, 0.82)	0.48 (0.17, 0.82)
Describing	−0.22 (0.24, 0.75)	0.05 (0.09, 0.75)	−0.80 (0.34, 0.75)	0.91 (0.13, 0.75)
Acting with awareness	0.11 (0.26, 0.63)	−0.04 (0.11, 0.63)	−0.91 (0.17, 0.63)	1.18 (0.21, 0.63)
Nonjudging	1.20 (0.17, 0.42)	−0.20 (0.12, 0.42)	−1.00 (0.14, 0.42)	1.23 (0.15, 0.42)
Nonreactivity	−1.07 (0.28, 0.64)	0.20 (0.13, 0.64)	−0.81 (0.16, 0.64)	0.85 (0.16, 0.64)
Mindfulness facets (*unstandardized* scores)				
Observing	2.40 (0.16, 0.36)	3.20 (0.06, 0.36)	2.80 (0.14, 0.36)	3.41 (0.11, 0.36)
Describing	3.07 (0.19, 0.49)	3.28 (0.08, 0.49)	2.60 (0.27, 0.49)	3.98 (0.11, 0.49)
Acting with awareness	3.05 (0.17, 0.27)	2.96 (0.07, 0.27)	2.39 (0.11, 0.27)	3.75 (0.14, 0.27)
Nonjudging	3.99 (0.13, 0.25)	2.91 (0.09, 0.25)	2.30 (0.11, 0.25)	4.01 (0.12, 0.25)
Nonreactivity	2.15 (0.17, 0.24)	2.93 (0.08. 0.24)	2.31 (0.10, 0.24)	3.33 (0.10, 0.24)

*Note. SE* = standard error of the mean.

a Scores have been standardized so that positive values are above the mean and negative values are below the mean.

#### Comparison of Profiles Across Test and Validation Samples

The acting with awareness facet mean in the “nonjudgmentally aware” profile was significantly higher in the test sample compared with the validation sample, *t*(44) = 3.05, *p* = .004, *d* = 0.94. The nonreactivity facet mean in the “high mindfulness” profile was also significantly higher in the test sample compared with the validation sample, *t*(81) = 2.81, *p* = .006, *d* = 0.63. All other profile indicator comparisons across samples were nonsignificant.

### Relationships Between Profiles and Mindfulness-Related Variables

The equality of the means of mindfulness-related variables was tested across the four profiles in the test and validation samples. Table 5 presents the unstandardized mean scores across latent profiles on the SCS (total scale and subscale scores), BDI-II, age of onset of depression, and number of previous episodes of depression in the test sample and Table 6 presents this in the validation sample. Chi-square statistics for pairwise differences between profiles on mindfulness-related variables in both samples are given in the supplementary materials (Table S1[available online]).

**Table 5.** Mean Scores (Unstandardized) Across Latent Profiles on Mindfulness-Related Variables in the Test Sample (*n* = 343): Self-Compassion, Depression, Age of Onset of Depression, and Number of Previous Episodes of Depression.

	Profile 1: Very low mindfulness (*n* = 101)	Profile 2: High mindfulness (*n* = 32)	Profile 3: Moderate mindfulness (*n* = 193)	Profile 4: Nonjudgmentally aware (*n* = 17)	Overall chi-square test value (*df* = 3)
SCS total scale	2.16 (0.06)	3.15^a^ (0.17)	2.60^b^ (0.05)	2.88^a,b^ (0.18)	58.96*
Self-kindness	2.01 (0.08)	3.19^a^ (0.22)	2.57^b^ (0.06)	2.69^a,b^ (0.20)	48.37*
Self-judgement	1.99 (0.08)	2.99^a^ (0.21)	2.36^b^ (0.07)	2.87^a,b^ (0.25)	33.55*
Common humanity	2.46 (0.09)	3.51^a^ (0.23)	2.92^b^ (0.07)	3.22^a,b^ (0.25)	29.12*
Isolation	1.96 (0.08)	2.73^a,b^ (0.23)	2.44^a,c^ (0.07)	2.77^b,c^ (0.27)	27.62*
Mindfulness	2.53 (0.09)	3.68 (0.17)	2.97^a^ (0.06)	3.06^a^ (0.19)	41.36*
Overidentification	1.97 (0.08)	2.82^a,b^ (0.22)	2.38^a,c^ (0.07)	2.67^b,c^ (0.31)	24.15*
BDI-II	18.19 (1.23)	4.60^a^ (1.05)	9.59 (0.78)	5.26^a^ (1.66)	80.45*
Age of onset of depression	24.54^a^ (1.52)	21.66^a^ (2.10)	22.61^a^ (1.05)	24.81^a^ (3.14)	1.79; *p* = .617
Number of previous episodes of depression	5.62^a^ (0.38)	5.24^a^ (0.52)	6.64^a^ (0.45)	5.27^a^ (0.80)	4.23; *p* = .237

*Note.* BDI-II = Beck Depression Inventory–Second edition; SCS = Self-Compassion Scale; *df* = degrees of freedom. Standard errors are given in parentheses. Negatively phrased items in the SCS (from the self-judgement, isolation, and overidentification subscales) were reverse-scored prior to analysis. Means sharing a superscript in a row indicate that they are *not* significantly different from each other (*p* > .05), for example, for SCS-total, Profile 2 does not significantly differ from Profile 4, but there is a significant difference between Profile 2 and Profiles 1 and 3.

* *p* < .001.

**Table 6.** Mean Scores (Unstandardized) Across Latent Profiles on Mindfulness-Related Variables in the Validation Sample (*n* = 340): Self-Compassion, Depression, Age of Onset of Depression, and Number of Previous Episodes of Depression.

	Profile 1: Nonjudgmentally aware (*n* = 29)	Profile 2: Moderate mindfulness (*n* = 204)	Profile 3: Very low mindfulness (*n* = 56)	Profile 4: High mindfulness (*n* = 51)	Overall chi-square test value (*df* = 3)
SCS total scale	2.35^a^ (0.13)	2.52^a^ (0.04)	1.97 (0.07)	3.22 (0.12)	94.87*
Self-kindness	1.98^a^ (0.17)	2.46 (0.06)	1.90^a^ (0.10)	3.33 (0.15)	69.90*
Self-judgement	2.76^a^ (0.19)	2.24 (0.06)	1.67 (0.08)	2.99^a^ (0.15)	85.07*
Common humanity	2.17^a^ (0.19)	2.96 (0.08)	2.37^a^ (0.13)	3.34 (0.16)	35.57*
Isolation	2.77^a^ (0.19)	2.19 (0.06)	1.87 (0.09)	2.90^a^ (0.15)	48.00*
Mindfulness	2.17^a^ (0.18)	2.99 (0.06)	2.38^a^ (0.11)	3.54 (0.15)	58.09*
Overidentification	2.27^a^ (0.17)	2.27^a^ (0.06)	1.68 (0.08)	2.98 (0.13)	74.23*
BDI-II	12.53^a^ (1.89)	11.37^a^ (0.81)	19.82 (2.00)	4.49 (1.02)	61.08*
Age of onset of depression	23.33^a^ (1.97)	22.53^a^ (0.88)	24.13^a^ (2.09)	26.67^a^ (2.88)	1.82; *p* = .611
Number of previous episodes of depression	6.39^a^ (0.75)	5.90^a^ (0.45)	6.19^a^ (0.68)	7.33^a^ (1.32)	1.24; *p* = .743

*Note.* BDI-II = Beck Depression Inventory–Second edition; SCS = Self-Compassion Scale; *df* = degrees of freedom. Standard errors are given in parentheses. Negatively phrased items in the SCS (from the self-judgement, isolation, and overidentification subscales) were reverse-scored prior to analysis. Means sharing a superscript in a row indicate that they are *not* significantly different from each other (*p* > .05), for example, for SCS-total, Profile 1 does not significantly differ from Profile 2, but there is a significant difference between Profile 1 and Profiles 3 and 4.

* *p* < .001.

#### Self-Compassion

##### Test sample

The “high mindfulness” group had significantly higher mean scores on the SCS total scale, and self-kindness, self-judgement, and common-humanity subscales, compared with all profiles apart from the “nonjudgmentally aware” group. The “high mindfulness” group also had a significantly higher mean score on the mindfulness subscale compared with all other profiles. For scores on isolation and overidentification subscales, the “high mindfulness” profile did not significantly differ compared with “nonjudgmentally aware” and “moderate mindfulness” groups. In contrast, the “very low mindfulness” group had a significantly lower mean score on the SCS total scale and all subscales compared with all other profiles. Taken together, these findings suggest that the “high mindfulness” and “nonjudgmentally aware” groups appeared to be the most adaptive in terms of self-compassion, and the “very low mindfulness” group the least adaptive.

##### Validation sample

The “high mindfulness” group had significantly higher mean scores on the SCS total scale, and self-kindness, common-humanity, mindfulness, and overidentification subscales, compared with all other profiles. The “moderate mindfulness” group was intermediate, and had significantly higher mean scores compared with the “very low mindfulness” group and significantly lower mean scores compared with the “high mindfulness” group on all SCS outcomes. The “nonjudgmentally aware” had significantly higher mean scores on total SCS compared with the “very low mindfulness” group and significantly lower mean scores compared with the “high mindfulness” group, but did not significantly differ compared with the “moderate mindfulness” profile. The relationship between the “nonjudgmentally aware” profile and individual SCS subscales were varied; although this profile did not significantly differ compared with the “high mindfulness” group in self-judgement and isolation scores, scores on self-kindness, common humanity, and mindfulness did not significantly differ compared with the “very low mindfulness group.” These findings suggest that the “high mindfulness” group was the most adaptive in terms of self-compassion, and the “very low mindfulness” group the least adaptive.

#### Depression

##### Test sample

The “very low mindfulness” profile had a significantly higher mean BDI-II score compared with all other profiles. The “high mindfulness” and “nonjudgmentally aware” profiles did not differ significantly, but had significantly lower BDI-II scores compared with other profiles. The “moderate mindfulness” profile differed significantly (in either a positive or negative direction) from all other profiles, having lower levels of depression than the “very low mindfulness” group and higher mean levels than the “high mindfulness” and “nonjudgmentally aware” profiles. These findings suggest that the “high mindfulness” and “nonjudgmentally aware” groups appeared to be the most adaptive in terms of depressive symptoms, and the “very low mindfulness” group the least adaptive.

##### Validation sample

As in the test sample, the “very low mindfulness” profile had a significantly higher mean BDI-II score compared with all other profiles. The “high mindfulness” profile had the lowest BDI-II score compared with other groups. The “moderate mindfulness” and “nonjudgmentally aware” profiles did not differ significantly, but differed significantly (in either a positive or negative direction) from other profiles, having lower levels of depression than the “very low mindfulness” group and higher mean levels compared with the “high mindfulness” profile. These findings suggest that the “high mindfulness” group appeared to be the most adaptive in terms of depressive symptoms, and the “very low mindfulness” group the least adaptive.

#### Age of Onset of Depression

For both test and validation samples, profiles did not significantly differ from each other in relation to age of onset of depression; overall chi-square tests and chi-square tests comparing pairs of profiles were nonsignificant.

#### Number of Previous Episodes of Depression

Mean number of previous episodes of depression did not significantly differ across profiles in both test and validation samples; overall chi-square tests and chi-square tests comparing pairs of profiles were nonsignificant.

## Discussion

Previous research studies in nonclinical populations have identified four FFMQ latent profiles: high mindfulness, low mindfulness, judgmentally observing, and nonjudgmentally aware groups. These profiles appear to relate in meaningful ways to measures of psychological well-being and be observed in those with and without meditation experience (Bravo et al., 2016; Pearson et al., 2015). We examined whether the same latent profiles were observed in a sample comprising people with a history of recurrent depression (Kuyken et al., 2015; J. M. G. Williams, Crane, et al., 2014). LPA in test and validation samples revealed that two of the four response profiles identified broadly mapped on to those found in the previous nonclinical samples. These groups were labelled “high mindfulness” (9.33% of the test sample, 15% of the validation sample) and “nonjudgmentally aware” (4.96% of the test sample, 8.53% of the validation sample) groups. In addition, a further two subgroups, “moderate mindfulness” (56.27% of the test sample, 60% of the validation sample), and “very low mindfulness” (29.45% of the test sample, 16.47% of the validation sample) were observed. Two previously identified profiles, “judgmentally observing” and “low mindfulness” (Bravo et al., 2016; Pearson et al., 2015) were not observed in either the test or validation sample.

We found largely similar profile associations with mindfulness-related constructs across test and validation samples. In both samples, the “high mindfulness” group was found to be the most adaptive in terms of depression and self-compassion and the “very low mindfulness” group was found to be the least adaptive. In the test sample, the “nonjudgmentally aware” group did not significantly differ compared with the “high mindfulness” group in terms of depression and almost all the self-compassion outcomes, supporting previous findings highlighting these two profiles as the most adaptive (Bravo et al., 2016; Pearson et al., 2015). In the validation sample, the “nonjudgmentally aware” group was less comparable with the “high mindfulness” group; they significantly differed on all outcomes except for two self-compassion outcomes (self-judgment and isolation). The greater difference between these two profiles in the validation sample could be attributed to the finding that mean acting with awareness in the “nonjudgmentally aware” profile was significantly smaller in the validation sample compared with the test sample.

Another point to note is that no associations were observed between latent profile membership and either age of onset of depression, or number of previous episodes of depression. These findings suggest that, within a sample of individuals with a history of highly recurrent major depression, group membership was not associated with severity of clinical course, as indexed by these variables. However, it remains to be seen whether associations would be observed between group membership and subsequent clinical course in individuals with a first onset of depression, or within a more heterogeneous sample with lower overall risk of relapse.

These findings highlight several issues. First, in participants with a history of recurrent depression, few individuals in both test and validation samples (less than 10%) had uneven profiles on the FFMQ, characterized by relatively high scores on some facets and low scores on others, yet the uneven profile identified within this smaller group (i.e., “nonjudgmentally aware”) replicated previous research. This pattern of findings is generally in line with the view of mindfulness as a coherent higher order construct and, at the same time, indicates that facet profiles can offer meaningful differentiations. As might be expected given the clinical history of our participants, the majority fell into the very low and moderate mindfulness groups. For those working clinically using mindfulness-based interventions, these findings are reassuring. They suggest that gauging participants’ mindfulness skills at entry to treatment using an overall score on the FFMQ is likely to provide a valid assessment of mindfulness skills in most cases. Additionally, they suggest that although individuals have different starting points, interventions designed to enhance mindfulness skills can reasonably adopt a progressive approach in which skills are targeted and developed in a way that makes most theoretical sense, rather than instructors being unduly concerned that individuals will have markedly different profiles that might suggest the need for a variety of different approaches to integrate areas of strength and weakness.

Second, in this sample, all of whom had a history of recurrent depression, a small number of individuals (9.33% of the test sample, 15% of the validation sample) had relatively high levels of mindfulness across all facets. Given that a significant relationship is typically observed between indicators of psychological well-being and levels of dispositional mindfulness (Baer et al., 2006), the significance of this subgroup is unclear. These individuals, while identified as being at high risk of recurrence of depression based on their clinical history, nevertheless reported fewer concurrent residual symptoms of depression and higher levels of self-compassion. If it is assumed that dispositional mindfulness is stable and trait-like, one possibility is that these participants reflect a subgroup whose depression has a distinct etiology, or in whom previous treatments or experiences have led, over time, to the development of greater levels of dispositional mindfulness, and perhaps also lower levels of ongoing vulnerability. On the other hand, if it is assumed that, at least in individuals who have not engaged in mindfulness practices, the capacity to relate to experience mindfully is quite fluid, it is equally possible that these participants have simply been assessed at a time of more complete remission, with lower levels of residual depressive symptoms (and concurring processing biases), and as such have a greater capacity for mindfulness. It would be instructive in future research to examine whether the high levels of dispositional mindfulness observed in such subgroups do in fact translate into better long-term outcome in the absence of clinical intervention. Given the relatively small proportion of individuals with recurrent depression who display this profile any such study would likely depend on the deliberate selection and inclusion of participants with these characteristics to ensure sufficient power. Equally, examining latent profiles in participants assessed during an episode of major depression, alongside the change in latent profiles as individuals move from wellness to illness, or from illness to recovery, has the potential to provide important information on the role of mindfulness in this disorder.

### Limitations

There are a number of limitations that should be considered in the interpretation of the results. First, previous research using LPA to explore subgroups defined on the basis of scores on the FFMQ has focused on college student samples (Bravo et al., 2016; Pearson et al., 2015) and the current study identified a four-profile solution in participants with a history of depression that shared some commonalities with the profiles identified in college students, but also had distinct features. However, we cannot conclude that the configuration of profiles identified in this study is specific to individuals with depression. For example, these profiles might also be observed in other clinical groups, or indeed other more demographically heterogeneous samples. Our sample was also disproportionately female and White, potentially limiting the generalizability of the findings, and differed from Bravo et al. (2016) and Pearson et al.’s (2015) samples in ways other than clinical history and nationality (e.g., age, education level), which may have contributed to differences between current and previous findings.

Second, we used a broad range of fit indices to determine the better fitting model in each sample and on the whole, concluded that the four-profile model appeared to be the optimal solution in both test and validation samples. However, fit indices did not consistently favor this model. For example, in the validation sample, the LMR-LRT favored the two-profile model, the BLRT favored the five-profile model, and the sBIC and AIC indicated preference for a model containing more than seven profiles. This could be attributed to variation in the performance ability of fit indices and we settled on the four-profile solution in both samples based on research demonstrating that the BIC outperformed other information criterion indices (Nylund et al., 2007) and superior classification probabilities in the four-profile solution compared with competing solutions. Nevertheless, the current findings require replication. Slight differences between test and validation samples in profile indicators (e.g., significantly higher acting with awareness in the “nonjudgmentally aware” profile for the test sample compared with the validation sample) and relationships between profiles and mindfulness-related variables (e.g., “nonjudgmentally aware” group being similarly as adaptive as the “high mindfulness” group in the test sample, but not in the validation sample) also underscore the need for current findings to be replicated.

Third, the cross-sectional nature of the study precludes conclusions regarding the temporal ordering of the relationships between profiles and mindfulness-related variables (residual depressive symptoms and self-compassion). A longitudinal design (e.g., examining transitions in profile membership over the course of a mindfulness-based intervention) would provide a stronger test of whether profiles (and changes in profile membership) causally account for differences across psychological variables (and changes in these variables over the course of intervention).

Fourth, while there is some overlap between our profiles and those of Pearson et al. (2015) and Bravo et al. (2016), this article does not explore whether the FFMQ functions in the same way in this U.K. sample with a clinical history as in the North American college student samples reported in these two previous articles. For example, we have not explored differential item functioning or measure invariance. Therefore, we cannot rule out the possibility that the measure is functioning slightly differently in the current sample, contributing to differences in latent profiles observed.

Finally, we were limited in this study by the small number of psychological variables that were shared across the two constituent trials from which the data derived. Similarly, the fact that the “nonjudgmentally aware” profile comprised small proportions of the samples meant that it was not possible to meaningfully explore the relationship between this profile and a broader range of psychological variables in each separate trial population independently. As a result, future research examining the relationship between the identified profiles and a broader range of outcomes to further validate and distinguish profiles would be very welcome, in particular, in relation to the profile that appears to reflect a pattern of co-occurring psychological characteristics which would not be readily captured by the use of the FFMQ as a continuous measure.

### Future Directions

As noted earlier, previous studies of interactions between FFMQ facets have shown that relationships with external indicators of psychological functioning vary with relative scale elevations on the FFMQ, particularly for the observing and nonreactivity facets (e.g., Desrosiers et al., 2014; Eisenlohr-Moul et al., 2012; Tomfohr et al., 2012). These findings suggest that the study of participants with uneven profiles might contribute to understanding the relationship between mindfulness and other variables, and the initial LPA studies with student samples seem to confirm this. In contrast, the FFMQ latent profiles we identified in our clinical sample appear to be relatively flat for most participants, suggesting that relatively few individuals have very high levels of one facet (e.g., observing) and simultaneously very low levels of another facet (e.g., nonjudgment, nonreactivity): profiles that would seem, on the basis of these previous studies, to be those that might be particularly informative for understanding nuanced associations between dispositional mindfulness and outcomes of interest. However, our findings do not rule out the possibility that variations in a particular facet might be more predictive of a maladaptive outcome when occurring in the context of some latent profiles rather than others. For example, hypothetically, variations in nonreactivity might be more predictive of relationship functioning for those in the “very low mindfulness” latent profile, than for those in the “high mindfulness” latent profile—despite the fact that levels of relationship functioning may be greater, overall, in the “high mindfulness” group. Examining such associations is a question for further work and has the potential to provide interesting insights into the way in which particular mindfulness skills support adaptive functioning in different broader dispositional contexts.

## Conclusions

Research exploring the potential utility of LPA as a tool to investigate the nature and correlates of mindfulness skills is in its early stages. On the basis of the findings of the current study, it remains unclear to what extent LPA adds significantly to the understanding of mindfulness in individuals with recurrent depression that might be obtained from the treatment of mindfulness, as assessed by the FFMQ, as a single higher order construct. However, this study extends previous finding to a population not previously studied with LPA. In addition, the study identified an interesting subgroup of participants who, despite having a clinical history of severe recurrent depression, nevertheless reported high levels of mindfulness. Longitudinal research is required to investigate whether the FFMQ profiles identified here change with treatment or as participants shift between depressive episodes and periods of wellness, and what the implications of high levels of dispositional mindfulness might be for the longer term outcomes of participants with a history of severe recurrent depression.

## Supplementary Material

Supplementary material
