# Supplementary material for: Latent Profile Analysis of the Five Facet Mindfulness Questionnaire in a Sample With a History of Recurrent Depression
Source: Assessment. 2017 Jun 19;27(1):149–63. doi: 10.1177/1073191117715114 (PMC6906539; doi:10.1177/1073191117715114)
Supplement: Supplementary material [file Supplementary_Materials_Table_S1.pdf]

Supplementary Materials for: Latent Profile Analysis of the Five Facet Mindfulness Questionnaire in a Sample with a History of Recurrent Depression

Table S1.
Chi-square test statistics for pairwise differences between profiles in the test sample (*N* = 343) and validation sample (*N* = 340).

Mindfulness-related variable	Profile comparison	Chi-square test statistic, *p*-value Test sample	Validation sample
SCS total scale	1 (very low mindfulness) vs. 2 (high mindfulness)	30.25, $p < .001$	86.62, $p < .001$
	1 vs. 3 (moderate mindfulness)	29.38, $p < .001$	38.64, $p < .001$
	1 vs. 4 (non-judgmentally aware)	14.00, $p < .001$	6.90, $p = .009$
	2 vs. 3	8.67, $p = .003$	29.40, $p < .001$
	2 vs. 4	1.15, $p = .283$	23.84, $p < .001$
	3 vs. 4	2.09, $p = .148$	1.43, $p = .233$
SCS self-kindness	1 vs. 2	26.43, $p < .001$	64.78, $p < .001$
	1 vs. 3	26.30, $p < .001$	18.75, $p < .001$
	1 vs. 4	9.98, $p = .002$	0.18, $p = .673$
	2 vs. 3	6.65, $p = .010$	26.29, $p < .001$
	2 vs. 4	2.74, $p = .098$	33.32, $p < .001$
	3 vs. 4	0.30, $p = .581$	6.47, $p = .011$
SCS self-judgement	1 vs. 2	20.20, $p < .001$	62.74, $p < .001$
	1 vs. 3	10.88, $p = .001$	27.29, $p < .001$
	1 vs. 4	11.31, $p = .001$	27.95, $p < .001$
	2 vs. 3	7.27, $p = .007$	19.98, $p < .001$
	2 vs. 4	0.12, $p = .734$	0.83, $p = .361$
	3 vs. 4	3.73, $p = .053$	6.59, $p = .010$
SCS common humanity	1 vs. 2	18.82, $p < .001$	23.34, $p < .001$
	1 vs. 3	12.76, $p < .001$	12.85, $p < .001$
	1 vs. 4	8.17, $p = .004$	0.70, $p = .403$
	2 vs. 3	5.61, $p = .018$	4.25, $p = .039$
	2 vs. 4	0.77, $p = .381$	20.98, $p < .001$
	3 vs. 4	1.25, $p = .263$	13.78, $p < .001$
SCS isolation	1 vs. 2	10.15, $p = .001$	36.30, $p < .001$
	1 vs. 3	16.66, $p < .001$	7.12, $p = .008$
	1 vs. 4	7.90, $p = .005$	18.99, $p < .001$
	2 vs. 3	1.37, $p = .243$	18.19, $p < .001$
	2 vs. 4	0.01, $p = .919$	0.28, $p = .599$
	3 vs. 4	1.32, $p = .251$	8.54, $p = .003$
SCS mindfulness	1 vs. 2	36.11, $p < .001$	41.57, $p < .001$
	1 vs. 3	13.92, $p < .001$	20.07, $p < .001$
	1 vs. 4	6.31, $p = .012$	1.02, $p = .314$
	2 vs. 3	13.72, $p < .001$	10.58, $p = .001$
	2 vs. 4	5.80, $p = .016$	33.69, $p < .001$
	3 vs. 4	0.20, $p = .656$	18.17, $p < .001$
SCS over-identification	1 vs. 2	13.07, $p < .001$	67.61, $p < .001$

	1 vs. 3	12.20, *p* < .001	28.58, *p* < .001
	1 vs. 4	4.75, *p* = .029	9.83, *p* = .002
	2 vs. 3	3.15, *p* = .076	20.71, *p* < .001
	2 vs. 4	0.14, *p* = .706	10.20, *p* = .001
	3 vs. 4	0.80, *p* = .373	0.001, *p* = .977
BDI-II	1 vs. 2	72.81, *p* < .001	47.80, *p* < .001
	1 vs. 3	28.55, *p* < .001	12.93, *p* < .001
	1 vs. 4	38.82, *p* < .001	6.97, *p* = .008
	2 vs. 3	12.51, *p* < .001	24.20, *p* < .001
	2 vs. 4	0.11, *p* = .737	13.20, *p* < .001
	3 vs. 4	5.30, *p* = .02	0.30, *p* = .584
Age of onset of depression	1 vs. 2	1.27, *p* = .261	0.52, *p* = .471
	1 vs. 3	0.87, *p* = .352	0.42, *p* = .519
	1 vs. 4	0.006, *p* = .937	0.08, *p* = .781
	2 vs. 3	0.14, *p* = .704	1.70, *p* = .192
	2 vs. 4	0.69, *p* = .405	0.86, *p* = .355
	3 vs. 4	0.42, *p* = .515	0.13, *p* = .718
Number of previous episodes of depression	1 vs. 2	0.37, *p* = .546	0.60, *p* = .438
	1 vs. 3	2.34, *p* = .126	0.13, *p* = .721
	1 vs. 4	0.16, *p* = .693	0.04, *p* = .841
	2 vs. 3	3.53, *p* = .060	1.00, *p* = .318
	2 vs. 4	0.001, *p* = .973	0.36, *p* = .551
	3 vs. 4	2.11, *p* = .146	0.35, *p* = .552

Note. Negatively-phrased items in the SCS (from the self-judgement, isolation, and over identification subscales) were reverse-scored prior to analysis. BDI-II = Beck Depression Inventory; SCS = Self-Compassion Scale.
